# Echoes of the Dallas Meeting

**Published:** 1910-06

**Authors:** 


### EDITORIAL DEPARTMENT.

This issue completes the twenty-fifth year of the "Red Back," and she is "doing pretty well, I thank you."

### ECHOES OF THE DALLAS MEETING.

The meeting of the Texas State Medical Association at Dallas, May 10-12 (ult.), was the largest in the history of that body. Over eight hundred members registered, and there were many visitors, not members, and many ladies, present. There were ninety odd delegates. The program was unusually full and varied, while the social entertainment was of high order and greatly enjoyed. Dallas is the place to meet, and instead of our "boxing the compass," and swinging around a circle whose radius extends from the gulf to the Colorado State line, we should locate at Dallas and meet there annually.

Somebody, six weeks in advance of the meeting, gave out to the press and it appeared under scare headlines, a declaration that at the approaching meeting a denunciatory resolution would be passed bitterly condemning the Governor for not approving certain legislation secured by the efforts of the Association. No one seems to know who was the author of the resolution, nor who had the unparalleled assurance to declare publicly through the daily press, six weeks in advance, *what* the Association would or would not do, but the telegram was from San Antonio. Well, the resolution never saw daylight. It didn't materialize. It was there, all right, but was referred to a committee and laid on the table,—out of respect to the medical profession—not so much for the *Governor,* because the better element of the profession opposed it and were not willing to be made the laughing stock of the State by doing so silly a thing as to pout and vent impotent spite because of disappointment, and we were, all, disappointed. It would have been beneath the dignity of a body of supposed to be learned, ethical, scientific gentlemen.

How-be-it, the President, Dr. W. B. Russ, of San Antonio, in his address before a mixed audience, lay and professional, condemned the Governor in strong language,—and his harangue was largely devoted to recounting the exploits of the Octopus, the actions of ye Council of Pharmacy, and denouncing all who are opposed to boss rule, dictation from Chicago, medical miscegenation and machine rule in our House of Delegates, to say nothing of the persistent disfranchisement of ninety-five per cent of members. Not a question was ever put before the general session so far as I know or ever heard.

The Daniel and the O'Farrell amendments to the Constitution, and the resolutions looking to certain changes proposed by Dr. Daniel and mentioned in the May "Red Back," were all sidetracked —referred to a committee named by ye President (Russ), and, of course, they went under. Ye President expended much energy in his address in denouncing all opponents of the Simmons-McCormack-Chase-Cantrell-Russ machine methods, and impugning the motives of the three Texas medical journals, accusing them of being in league with enemies of organization, a charge that is as contemptible as it is false, and known to be false by all who are familiar with the work of these journals for many, many years. In brief, Dr. Russ opened up the old sore and poured coal oil on the slumbering fires of discontent. He "made a flat." He was not backed up in his assaults upon the opponents of machine rule, etc., nor his vindictive and discourteous attack upon the Governor; and when he realized that he had overshot the mark and that the general sentiment in the House was not in sympathy with his radical talk, he endeavored—in the House—to hedge, and made a great show of keeping down the threatened damnation of Governor Campbell, which had been heralded from San Antonio as coming, six weeks in advance of the meeting. He made another flat. The resolution was not even offered in the House. It was suppressed, laid on the table, at the solicitation of those who are proud of the record, traditions and achievements of the profession in the past, and jealous of its dignity. I give part of the newspaper report of Dr. Russ' speech in the church, side by side with his remarks in the House, that readers may see what an adept our late President is in blowing hot and cold, in swapping sides, and going back on himself. Bah!

A most remarkable incident occurred at the banquet. There were perhaps a hundred or more ladies and young misses, and as many gentlemen, seated at the elegant supper when the game was opened. Dr. C. E. Cantrell is an ex-President, a member of the "Ancient and Honorable Board of Selectmen,"—no, trustees—of the Octopus, and Poo-bah of the Texas House of Delegates. I may say, Chauffeur of the Machine (which is in splendid working order). He was called on by the toastmaster, Dr. McReynolds, to respond to a toast to the A. M. A. He immortalized himself by giving the ladies some most excellent advice, advice that doubtless will save many babies from blindness. He said in effect: "Ladies, when your baby is born, *as soon as it comes,* wash its eyes and drop in some nitrate of silver—in case the eyes may be infected." Some of the members had half-grown daughters present who doubtless enjoyed it immensely. Stiles talked hookworm, and a Chicago doctor lectured on brain surgery in response to a toast to him. It reminded me of the fox's supper given to the stork. He served thin soup in shallow plates. The stork didn't get any. A lady near me remarked that ice cream didn't taste good with sore eyes and hookworm gravy, and shoved her plate aside.

I must qualify the above a little. The resolution making the annual dues $1.00 and the subscription to the Association's journal $1.00 and optional, will go into effect. It is a case of must. The Trustees were instructed to correspond with the Postoffice Department looking to bringing the journal into accord with the requirements of the law. The resolution to publish the papers separately in a bound volume I withdrew, seeing that the body of the membership was to be ignored, as usual, not given a vote, and the amendment to the Constitution was reported by the committee to be unnecessary, inasmuch as the ground is covered by the constitution of the county societies, the entering portals of the State Association.

This committee reported in the afternoon of the last day, thus making a vote by the ninety-five per cent of the members impossible. Can such things be? Will they be longer tolerated? I answer, yes. They will submit to anything for fear of the lash;—they will be called *scabs* if they do not.

Amarillo was voted next year's convention and the following officers were elected: President, Dr. John T. Moore, of Houston; First Vice-President, Dr. A. B. Reynolds, of Stamford; Second Vice-President, Dr. W. A. Harper, of Austin; Secretary, Dr. Holman Taylor, of Fort Worth; Treasurer, Dr. C. A. Smith, of Texarkana.

Councilors: Third District, Dr. D. R. Fly, of Amarillo; Fifth District, Dr. W. A. King, of San Antonio; Sixth District, Dr. W. J. Hamilton, of Laredo; Ninth District, Dr. James A. Hill, of Houston; Twelfth District, Dr. A. C. Scott, of Temple; Fifteenth District, Dr. L. G. Turner, of Daingerfield.

Trustee: Dr. W. R. Thompson, of Fort Worth.

Delegates to American Medical Association: Dr. W. B. Russ, of San Antonio, and Dr. J. S. Turner, of Dallas. Alternates: Dr. O. L. Norsworthy, of Houston, and Dr. G. B. Foscue, of Waco.

The election of Dr. Jno. T. Moore to the presidency was hailed with delight. He is perhaps the most popular man in the society, a general favorite, and nicknamed "the Old Reliable." He is able, modest, fair, conservative and just. We look for harmony and the square deal under his administration. The machine can not control *him*. Dr. Moore has worked faithfully and efficiently as chairman of the Committee on Education and in many other positions, and is justly recognized as one of the best, truest and most useful men in the Association. He is a native of East Texas, an alumnus of our own Texas Medical College, and was late adjunct to the chair of Practice during the incumbency of the late Professor McLaughlin.

A few friends, by private subscription gotten up by Dr. Cantrell, bought Dr. Chase a watch. It was presented by Dr. S. R. Burroughs. Exit Chase. Enter Dr. Holman Taylor as Editor-Secretary and General Manager.

